# Movement Patterns and Diel Activity of *Anguilla japonica* in the Middle Part of a Large River in South Korea

**DOI:** 10.3390/ani10122424

**Published:** 2020-12-17

**Authors:** Jeong-Hui Kim, Sang-Hyeon Park, Seung-Ho Baek, Min-Ho Jang, Ju-Duk Yoon

**Affiliations:** 1 EcoResearch Incorporated, Gongju 32588, Korea; ragman-k@hanmail.net (J.-H.K.); psh@ecoresearch.co.kr (S.-H.P.); bsh@ecoresearch.co.kr (S.-H.B.); 2 Department of Marine Fisheries Resources, Mokpo National University, Mokpo 58554, Korea; 3 Department of Biology Education, Kongju National University, Gongju 32588, Korea; jangmino@kongju.ac.kr; 4 Restoration Center for Endangered Species, National Institute of Ecology, Yeongyang 36531, Korea

**Keywords:** acoustic telemetry, vertical movement, diel movement, nocturnal species

## Abstract

**Simple Summary:**

*Anguilla japonica* is an IUCN red list species facing a natural reduction in population due to environmental changes and artificial decline due to various human activities (disturbance in foraging, river, etc.). While many of these disturbances mainly occur in freshwater areas, studies on the ecology of *A. japonica* in the freshwater environment are limited compared to those of *Anguilla anguilla* and *Anguilla rostrata*. Freshwater is an important habitat for eels to grow to the spawning period. With insufficient numbers of adults supplied to the sea for spawning, eels may eventually become extinct. This study aimed to provide ecological information of the continental phase in the freshwater ecosystem by examining diel activity along with the movement patterns of eels in the pure freshwater environment based on the results of monitoring. In the future, it is necessary to establish a protection and management strategy for the conservation of eels living in rivers based on the results.

**Abstract:**

To investigate movement patterns and diel activities of *Anguilla japonica* in the freshwater ecosystem, we applied acoustic telemetry on *A. japonica* in the Geum River, a large river in South Korea. The acoustic tags were attached on 19 individuals of *A. japonica* (12 with a depth sensor) in May and October 2015 and tracked at approximately 100-km sections from an estuary barrage by 20 automatic listening stations. Only four individuals showed longitudinal movement (mean, 5.2 km), and others were detected by the receivers at release sites; therefore, *A. japonica* showed high site fidelity. We did not identify seaward migration during the study period (May to November). Conversely, *A. japonica* showed active diel movement. The number of detections (*p* = 0.002) and movement distance (*p* = 0.004) were higher at night-time (18:00–06:00). As most individuals were actively moving at nighttime, we confirmed that *A. japonica* is nocturnal, although few individuals also showed daytime movement. Although the population and habitats of *A. japonica* have been decreasing simultaneously, the East-Asian countries are still severely exploiting rivers and streams to use water resources, and result in habitat simplification generated. Therefore, these results contribute to effective *A. japonica* management regarding habitat and population conservation and restoration.

## 1. Introduction

Freshwater eel (genus *Anguilla*) is a catadromous fish, which spawns in the sea, but lives parts of its life in freshwater [1,2]. *Anguilla japonica*, which has moved to the sea for spawning, spawns near the Mariana Islands. When the eggs hatch, the leptocephalus larvae ride the North equatorial current and Kuroshio current and migrate to East Asia (South Korea, Japan, China, Taiwan, and northern Philippines) [3,4]. After metamorphogenesis into glass eels, they migrate to estuaries, coastal areas, and rivers to spend their lives. Some individuals inhabit rivers until their spawning migration [2].

According to the genetic study of a population of *A. japonica* in Asia, *A. japonica* in each country (Korea, Japan, China, and Taiwan) were found to belong to a single population [5,6,7]. The fishing rate of *A. japonica* has markedly declined since the 1970s [8]. Overharvesting, habitat destruction, migration restrictions because of instream structures such as dams and weirs [9], and climate change [10,11,12,13] are identified as main causes. When considering all the available data on its multiple life-history stages, *A. japonica* is estimated to have suffered at least a 50% population decline over a period of three generations (24 years). In the IUCN (International Union for Conservation of Nature and Natural Resources) Red List published by IUCN in 2014, *A. japonica* has been designated as an endangered species and is suggested to require management [14]. More recently, *A. japonica* was described as “endangered” on the Japanese Red List (published by the Ministry of Environment, based on the catch data of inland adult eels compiled by the Ministry of Agriculture, Forestry, and Fisheries).

Research on *A. japonica* mainly deals with specific areas. Joint research by multiple countries has revealed the spawning ecology [2,15,16] and migration of *A. japonica* [17,18] and environmental factors affecting its migration [19]. Most of the studies have been associated with spawning migration in the ocean; ecological studies in the freshwater ecosystem where the eels grow into yellow eels have been conducted on a limited basis in terms of their diet (e.g., Kaifu et al. [20], Wakiya and Mochioka [21]) and morphological changes [22].

The freshwater ecosystem is an important environment for maintaining the wild eel population. It is important to maintain and manage this population, as individuals grown sufficiently in the freshwater environment constitute a potential spawning population that can increase productivity. Studies have been conducted on the ecology of yellow eels in freshwater for *Anguilla anguilla* (European eel) (e.g., Piper [23,24], Verhelst et al. [25,26], Trancart et al. [27]) and *Anguilla rostrate* (American eels) (e.g., Béguer-Pon et al. [28], Kwak et al. [29]); nevertheless, studies on the migration of *A. japonica,* distributed in Asia, in freshwater are scarce.

The East-Asian countries have continuously transformed rivers and streams severely to use water resources and this results in habitat simplification of *A. japonica*. As the population and habitats of *A. japonica* have been decreasing simultaneously, urgent conservation is needed. To establish an efficient conservation strategy for eels, research on the migration, movement, and habitat selection should be preceded. In this study, the longitudinal and vertical movement of *A. japonica* was monitored by applying acoustic telemetry to the silver eels in the continental phase (middle part of large river). This study aimed to provide ecological information of the continental phase in the freshwater ecosystem by examining diel activity and vertical movement patterns of *A. japonica* in the pure freshwater environment based on the results of monitoring. Furthermore, we examined whether the artificial structures built in the river have an effect on the migration of *A. japonica*. The data presented through these results contribute to establishing a management strategy for maintaining the potential spawning population of *A. japonica* in a pure freshwater environment.

## 2. Materials and Methods

### 2.1. Study Site

**Figure 1:**
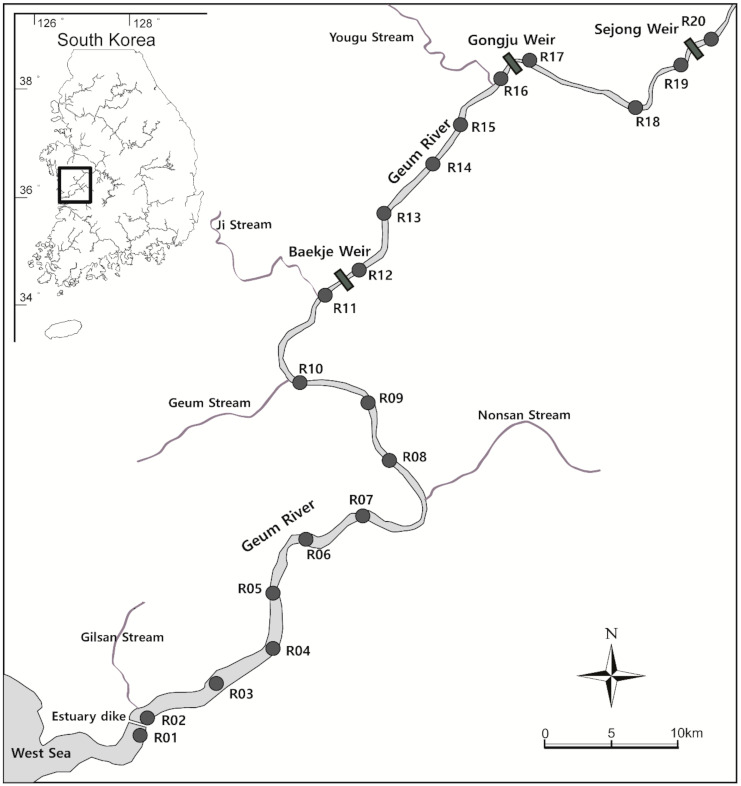
A map showing the study sites. Black circles are sites with installed VR2W acoustic receivers.

Monitoring of *A. japonica* movement was conducted in the midstream section of the Geum River, a major river in South Korea (Figure 1). The Geum River is a representative river inhabited by *A. japonica*; glass eels move through the Geum River estuary to a freshwater environment from December to April. An estuary bank and three large-scale weirs have been installed in the middle and downstream of the Geum River, affecting the longitudinal migration of many aquatic species. The mainstream of the Geum River where the survey was conducted had an average bottom width of 200 to 300 m and a depth of approximately 6 m with the riverbed mainly composed of clay and sand. It comprises a completely freshwater area with a salinity of 0 psu. Dredging was carried out in most of the sections for the Four Major Rivers Restoration Project from 2010 to 2012, and the river forms a ladder-shaped channel.

### 2.2. Acoustic Tagging

*A. japonica* was captured at the three collecting stations (R11, R16, and R19) located at the lower part of each three weirs in the Geum River using a fyke net. In order to capture enough numbers of *A. japonica*, the collection was conducted continuously for two weeks in May 2015 and October 2015, respectively, and as a result total 30 individuals of *A. japonica* were captured (R11, 18 individuals; R16, 5 individuals; R19, 7 individuals). Every individual was immediately transported to the laboratory after capture using the 100 L water tank with aerator. All collecting stations were located within 30 min of the laboratory, which was close enough to minimize the stress on *A. japonica* during transportation. After transportation, *A. japonica* was transferred into a water tank with an aerator installed (1 × 0.4 × 0.6 m in size), after that, the light was blocked in order to pacify *A. japonica*. After at least a week of acclimatization, healthy individuals with no apparent damage that swam well were selected and tagged. For the study, a transmitter (Thelma Biotel, Norway) was attached to 19 subjects; 12 subjects were fitted with a depth sensor (ADT-9, diameter 9 mm, length 34 mm, weight in the water 3.3 g) capable of monitoring depth movement, and nine subjects were fitted with a sensor capable of monitoring movement only (LP-7.3, diameter 7.3 mm, length 18 mm, weight in the water 1.2 g).

To attach the tag, the subjects were first transferred to an anesthesia tank and anesthetized using ethyl-3-aminobenzoate methanesulfonate salt (Sigma-Aldrich, Darmstadt, Germany). After anesthesia, the total length (mm) and body weight (g) were measured, and activated transmitters were surgically attached to the back of the *A. japonica* in front of the dorsal fin with nylon string. To prevent infection due to tag attachment, antibiotics (Kanamycin sulfate, Sigma-Aldrich) were injected into the muscle. The subject fitted with a tag recovered for more than 2 h in a water tank with sufficient supply of oxygen, and its behavior and appearance of infection were observed in the laboratory for a week. After confirming that there was no difficulty in swimming, the *A. japonica* were moved to each release point and released.

The mean total length of the *A. japonica* fitted with a transmitter was 484.0 ± 47.8 mm (± standard deviation) with a minimum total length of 410 mm (Table 1). The subjects were adults based on the standards set by Okamura et al. [30] and could endure the weight of the transmitter, as the transmitter weighed less than 1% of their body weight.

### 2.3. Acoustic Monitoring

For monitoring, 20 fixed receivers (VR2W, Vemco, Bedford, NS, Canada) were installed in the 100 km section from the middle to the downstream of the Geum River (Figure 1). The detection range of VR2W was approximately 200 m, and it was possible to cover the lower width of the mainstream of the Geum River within the survey section on site. The receivers were placed in the center of the channel if the river width exceeds 200 m. The distance between the receivers ranged from 1.3 km to 11.1 km, and receivers were installed at an average interval of 5 km. A receiver was placed in the vicinity of each weir to determine whether the *A. japonica* moved up and down the three weirs located within the survey section. Although there was a stream flowing into the Geum River within the survey section, a receiver was not installed for the inflow stream, excluding the movement to the stream from the scope of this study.

Twelve, three, and four subjects fitted with a tag were respectively released at each of the three points, R11, R16, and R19, located downstream of each weir. Based on data on the distribution of *A. japonica* in the Geum River, most subjects were released in the R11 area, the downstream area where most *A. japonica* live, and similar numbers were released at R16 and R19. To prevent data loss due to failure or loss of the receiver, the receiver was inspected, and the detected data were downloaded once every two weeks. The final analysis was conducted based on the data downloaded on 30 November 2015.

### 2.4. Data Analysis

The longitudinal movement of *A. japonica* was divided into movement boundary (km) and total movement distance (km). Movement boundary refers to the distance between the lowermost receiver and the uppermost receiver detected for each subject, and the total movement distance was obtained by calculating the cumulative movement distance between receivers. The vertical movement (m) was the total vertical movement distance of the *A. japonica* and this was analyzed using the monitoring results from the 12 subjects fitted with the depth sensor.

To compare the body size of the subjects with long-distance movement (between receivers) and those without movement, the Mann–Whitney U test, a nonparametric test, was used to compare the total length and weight of the two groups. The diel activity patterns were compared to determine the main activity time; for this, diel activities were divided into daytime activities (06:00–18:00) and nighttime activities (18:00–06:00). As the movement data on the day of release could affect the results of day and night movements, the data from the day of release were excluded from the analysis. Based on the obtained data, the number of detections during the daytime and nighttime were compared for each subject using the Wilcoxon signed-rank test, a nonparametric statistical test. Whether there was a difference in the average depth between daytime and nighttime was also analyzed using the Wilcoxon signed-rank test. These analyses were performed using SPSS v. 18.0 (SPSS Inc., Chicago, IL, USA).

## 3. Results

### 3.1. Longitudinal Movement

**Figure 2:**
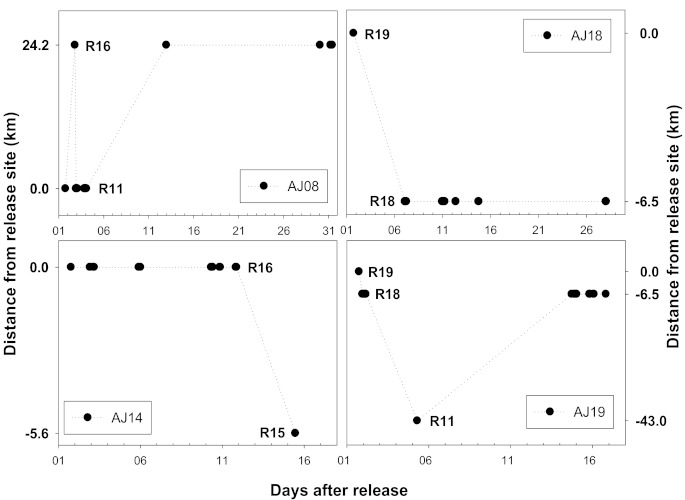
Migration pattern and distance of four *A. japonica* individuals that showed longitudinal movement. The plus value on the *y*-axis means movement toward an upstream direction, and the minus value means movement toward a downstream direction.

Most of the *A. japonica*, except for some individuals, were found to have stayed near the release area. Among the 19 individuals fitted with a transmitter, four individuals, AJ8, AJ14, AJ18, and AJ19, demonstrated long-distance movement (Table 2, Figure 2); they traveled a distance of 5.6 to 79.5 km within the 5.6 to 43 km boundary (the actual distance is believed to be even longer, as the data only included the signals detected by the receivers). Three of the four subjects that showed migration behavior were released in October, except for AJ14, and no subject identified was found to have moved through the estuary bank to the sea.

As a result of analyzing the movement of each individual *A. japonica* in which movement was detected, subject AJ8 reached the R16 point (24.2 km upstream) after repeating the up and down movement the day after its release; it was found to have traveled 72.6 km before the final signal was detected. The final signal from subject AJ14 was detected at 5.6 km downstream. Subjects AJ18 and AJ19 released at R19 also demonstrated longitudinal movement. Subject AJ18 moved 6.5 km downstream, and AJ19 passed through two weirs, demonstrating the most movement (79.5 km) within the widest boundary (43 km). There was no statistical difference between the subjects with movement (mean ± standard deviation; length 517.8 ± 71; weight 171.3 ± 92.3) and those without movement (length 484.7 ± 41.7; weight 137.3 ± 39.9) in terms of their overall length (Mann–Whitney U test, U = 28.5, *p* > 0.05) or body weight (Mann–Whitney U test, U = 27.0, *p* > 0.05). The AJ15 has not detected both longitudinal and vertical migration, which is believed to be due to the fact that the AJ15 entity has moved out of the receiver’s detection range within a short period of time after being released and stayed out of the detection range continuously.

### 3.2. Diel Activity

**Figure 3:**
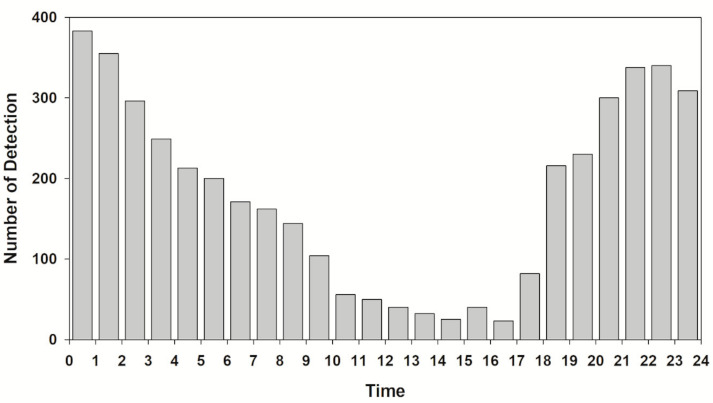
The number of diel detection patterns of tagged *A. japonica* in the Geum River.

Regarding the results of analyzing the number of detections by time, the total number of detections was usually within 50 from 10:00 to 17:00 during the week (56 times from 10:00 to 11:00), and it started to increase after 17:00 and showed numerous detections, more than 300, from 20:00 to 22:00 (Figure 3). Afterward, it showed a continuous decline, and the total number of detections decreased from 09:00 (Figure 3). Although there were some differences among individuals, most showed similar detection patterns.

**Figure 4:**
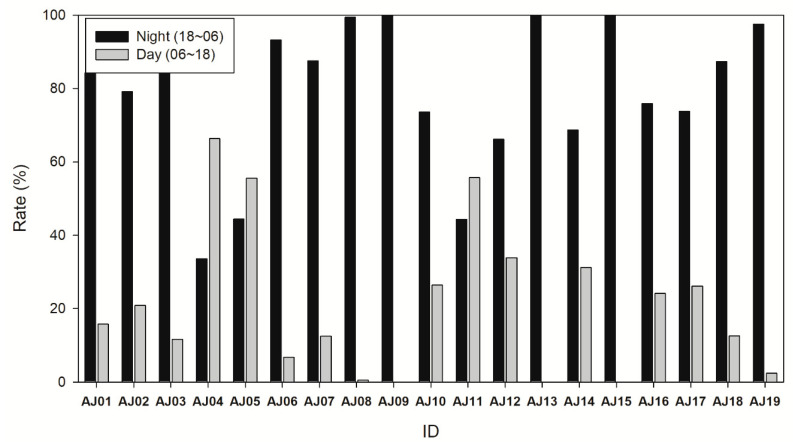
The proportion of detections during the nighttime (18:00–06:00) and daytime (06:00–18:00).

More signals were detected at night when comparing the number of detections during the day (6:00 to 18:00) and night (18:00 to 6:00) of each individual (Wilcoxon signed-rank test, Z = −3.159, *p* = 0.002). Except for three subjects (AJ4, 5, and 11), more signals were detected at night (Figure 4). For the total number of detections, the total number of signals started increasing at 17:00 (increase time) and decreasing at 9:00 (decrease complete).

### 3.3. Vertical Movement

**Figure 5:**
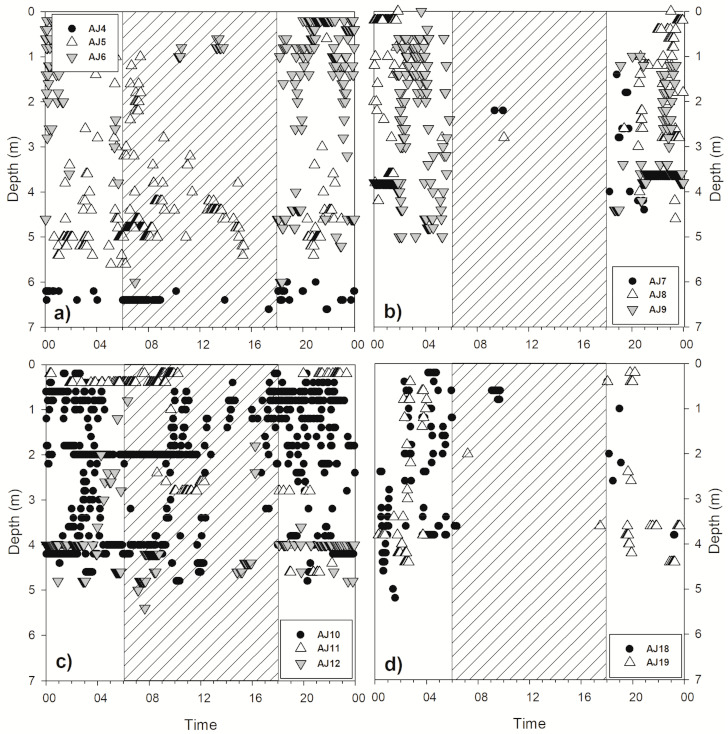
Average diel movement of 11 *A. japonica* fitted with a depth sensor; the diagonal line and white backgrounds represent nighttime and daytime based on average 12-h periods during 24 h, respectively. (**a**) AJ4, AJ5, AJ6, (**b**) AJ7, AJ8, AJ9, (**c**) AJ10, AJ11, AJ12, (**d**) AJ18, AJ19.

**Figure 6:**
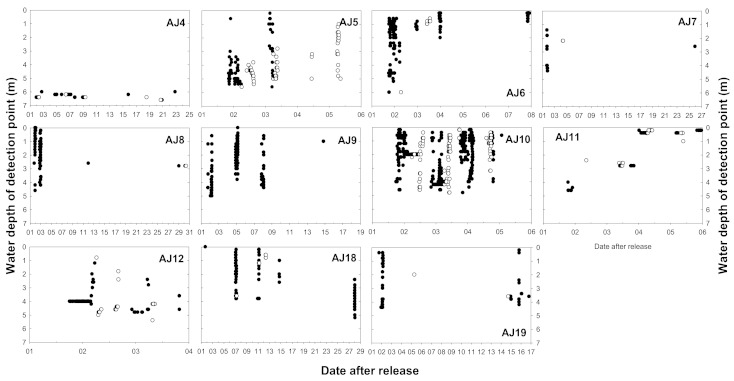
Daily movement patterns of 11 *A. japonica* fitted with a depth sensor: black and white circles indicate nighttime and daytime detection, respectively. AJ15 was excluded because there was no vertical movement.

As a result of analyzing the vertical movement patterns of 12 subjects fitted with a transmitter capable of measuring the depth, it was confirmed that they moved more vertically during the night than during the daytime (Table 2). The comparison of the total movement distance of each subject during the day and night revealed that vertical movement was frequently performed at night (Wilcoxon signed-rank test, Z = −2.845, *p* = 0.004). During the nighttime, although the average vertical travel distance differed for each subject, subject AJ10 moved an average of 49.7 m. Subjects AJ5 and AJ10 demonstrated vertical movement even during the daytime (Figure 5). Some subjects moved for approximately 1 to 2 h after sunrise (AJ5) and started vertical movement for approximately 1 to 2 h before sunset (AJ10). As the surveyed area had been evenly dredged to a depth of 6 m, the maximum depth of *A. japonica* movement was approximately 6 m. Except for AJ4 and AJ15 with small vertical movements, the *A. japonica* widely used the range from the water surface to the maximum depth (Figure 6). They showed no tendency to prefer shallow or deep areas when moving vertically at night, demonstrating a continuous random movement regardless of the vertical direction. After most subjects ceased activity, no signal was detected. Some subjects were active every day during the survey period, but most stopped for some time between their active periods.

## 4. Discussion

Among the 19 subjects fitted with a tag, 15 subjects (78.9%) were only detected by one receiver. As most of the subjects stayed in the area of release, and even the subjects demonstrating movement returned to the area of release, the *A. japonica* appeared to have high site fidelity. This is in line with the results of studies conducted on the site fidelity of *A. anguilla* and *A. rostrata* in the freshwater environment [28,31,32]. Homing behavior has been observed for both *A. anguilla* and *A. rostrata* [1,33]. Tesch [2] found that burrows and cavities were utilized as resting places and shelter for eels and studies have documented the fidelity of tagged eels to discrete refuges [34,35]. Walker et al. [36] demonstrated that estuarine eels return to the same site every night. The average distance between receivers installed in this study was 5 km (minimum 1.3 km, maximum 11.1 km) so it was difficult to confirm high site fidelity even if there was no result detected by other receivers except for one. However, the subjects monitored in this study are believed to have shown similar movement patterns even under the setup based on the results of the study by Verhelst et al. [26] (average receiver interval 1.9 km, average travel distance 4 km). The study by Itakura [37] also reported home ranges of 0.085 ± 0.068 km^2^, and linear home ranges of 744 ± 268 m, revealing that the home ranges of *A. japonica* were not large. In this study, the distance between receivers was rather long, resulting in limitations to present accurate results; however, *A. japonica* appeared to demonstrate the same movement pattern based on the results of the day and night signal detection and vertical movement. Although it was not possible to confirm their homing behavior, most of the subjects (including those that moved) continuously showed signals in the area where they first settled, and even the subjects that had moved out of the detection range of the receiver were often re-detected, suggesting their high site fidelity. However, some receivers missed the detection while some of the tagged individuals were passed apparently, since those receivers were placed in the path between two receivers which detected those individuals (AJ08, AJ19). Therefore, in future research using acoustic telemetry in freshwater, the method of receiver installation (number of receivers, location, and interval of receivers) should be complemented so that more clear results could be achieved.

In general, *A. japonica* living in South Korea migrate to the sea for spawning around October [38]. In this study, in order to confirm the movement during the spawning period, silver eels fitted with a transmitter (12 subjects) were released in October, but none of the subjects moved downstream and escaped the estuary bank. This indicated that no subject in the study performed spawning migration.

Weirs, dams, and estuary banks pose obstacles to the movement of eels, leading to a decrease in population and density (*A. anguilla*, White and Knights [39], Feunteun [40], *A. rostrata*, Verreault et al. [41], Hitt et al. [42], Kwak et al. [29]). In this study, subject AJ19 passed through a weir (Gongju and Baekje weirs, 6 m or more in height) located in the mainstream, supposedly through the fishway installed in the weir. Although eels demonstrate excellent physical ability by passing over a weir without using a fishway from time to time [43], it is difficult for them to pass through a large-scale weir. In South Korea, there are approximately 35,000 weirs installed in rivers and streams, but only 15% have a fishway [44]. There are also many estuary dams resulting in numerous problems for eels to migrate. Therefore, it is necessary to establish a plan to ensure the longitudinal connection of rivers to protect and preserve the number of eels.

*A. japonica*, similar to other species of genus *Anguilla*, is nocturnal [26,37,45,46] showing movement mainly at night (Figure 3). The number of detections increased from the time when the sun began to set, and the activity was highest until the late nighttime (20:00 to 3:00). In this study, the reason that the signals from the *A. japonica* disappeared during the daytime was suspected to be because they were hiding in a shelter or space that the hydroacoustic sonar could not reach. The nocturnal behavior was associated with foraging and predation avoidance. Eels feed on a variety of prey such as crayfish, crab, shrimp, bivalves, insects, and fish [20,21,47]. There is a difference in the preferred prey because prey distribution differs by habitat (freshwater vs. brackish water). Eels shift their diet according to the situation and generalize their food choices [48,49]. Eels capture prey by olfaction rather than visual cues [46,50]. The reason why they move more at night is believed to be that they wander around and capture any prey they encounter by chance instead of waiting for prey. Especially, eels exhibit behavioral characteristics of constantly exploring through the shallow and deep areas along the waterside, and the study by Jellyman and Sykes [45] also reported similar results. In other words, there is a high probability that the reason for their increased activity at night is because this is when they feed.

In studies analyzing the diel activities of *A. japonica*, *A. japonica* demonstrated a higher level of activity at night, but there were some cases of activities detected during the daytime (Figure 5). The daytime activity found in the present study possibly occurred mostly during overcast weather, as found by McGovern and McCarthy [35], or during periods of increased turbidity [32]. In this study, daytime movements showed similar patterns to the depth movement at night, suggesting that some subjects were foraging during the daytime. The study by Dou and Tsukamoto [51] conducted in a laboratory on glass eels confirmed daytime foraging activity of eels, and adult eels were also reported to perform daytime feeding [47]. In this study, the subjects that moved during the daytime showed vertical movement during the daytime and at night; therefore, the daytime movements appeared to have been performed out of necessity. Moreover, there was a possibility that it could have been because of a temporary disturbance in the habitat area rather than foraging activity. Itakura et al. [47] reported that daytime migration occurred because of light pollution. However, it does not seem to have a significant impact on the behavior of eels considering the nature of the eels suggested by their choice of hiding place that even made their signals disappear.

Information on how living organisms move and use their habitats is particularly important in establishing direct conservation strategies for them. In this study, the reason that the signals from the *A. japonica* disappeared during the daytime was suspected to be that they were hiding in a shelter or space that the hydroacoustic sonar could not reach. The studies by Jellyman and Sykes [45] and Verhelst et al. [26] also suggested the cause of their nocturnal behavior as a strategy to avoid predators. However, unlike the ecosystem in Europe, there are no fish predators in South Korea capable of predating eels underwater. Conversely, birds such as herons and egrets and some mammals such as otters could still have an impact, and the nocturnal behavior of eels could be a way to avoid these species.

In this study, *A. japonica* was found to move frequently along the shallow sections (waterside). The study by Jellyman and Sykes [45] also reported that eels mainly roamed near the banks and watersides of reservoirs. Therefore, it would be important to ensure diversity in the waterside for the conservation of eels. The development in the waterside may result in the loss of aquatic vegetation and structural diversity in the adjacent shallow areas, which could affect eels. In Japan, loss of bottom fish populations [52] was identified after a waterside construction, and there were positive correlations between the amount of shoreline revetment and declines in eel catches in 18 rivers and nine lakes [53]. In the United States, many studies have been conducted on the impact of urbanization and levee construction on the population of *A. rostrata*, and these were indeed identified as the cause of the decrease in the population [54,55]. In South Korea, as waterside maintenance and channelization are continuously carried out to prevent floods and disasters, it is necessary to establish management measures to avoid disturbing the habitat of *A. japonica* affecting their survival. With no clear plan for the conservation of *A. japonica* in South Korea, shoreline protection and waterside protection are important measures for protecting *A. japonica* habitats.

## 5. Conclusions

*A. japonica* is an IUCN red list species and designated and managed as a red list species in a specific country (Japan) that are endangered, facing a natural reduction in population due to environmental changes and artificial decline due to various human activities (disturbance in foraging, the river, etc.). Among the various conservation strategies for the conservation of Asian eels, development of habitat protection and management is the most important plan of action. However, East-Asian countries have continuously transformed rivers and streams to use water resources, and result in habitat simplification of aquatic organisms. The Asian eels favor habitats of high complexity and diversity which provide a lot of prey items and many hiding places. Thus, to improve the number of eels and habitat quality, habitat restorations through river renaturalization should be conducted. Freshwater is an important habitat for eels to grow before the spawning period. With insufficient numbers of adults supplied to the sea for spawning, eels can eventually become extinct. Therefore, it is necessary to make policies or laws to protect eel populations and freshwater habitats for the conservation of *A. japonica*.

## Figures and Tables

**Table 1:** Body size, release, and detection information of tagged *A. japonica* (*, depth sensor).

ID	Total Length (mm)	Body Weight (g)	Release Site	Release Date (yy-mm-dd)	Last Detected Date (yy-mm-dd)	Last Detected Site	Total Number of Detection	Number of Receivers Detected on
AJ1	550	221.6	R11	2015-05-30	2015-06-01	R11	19	1
AJ2	510	154.4	R11	2015-05-30	2015-06-09	R11	379	1
AJ3	510	147.7	R11	2015-05-30	2015-09-09	R11	782	1
AJ4 *	493	141	R11	2015-10-15	2015-11-05	R11	110	1
AJ5 *	478	128	R11	2015-10-15	2015-10-19	R11	270	1
AJ6 *	470	110	R11	2015-10-15	2015-10-21	R11	237	1
AJ7 *	465	102	R11	2015-10-15	2015-11-08	R11	32	1
AJ8 *	410	81	R11	2015-10-17	2015-11-16	R16	174	2
AJ9 *	450	117	R11	2015-10-17	2015-10-30	R11	2328	1
AJ10 *	475	109	R11	2015-10-17	2015-10-21	R11	1067	1
AJ11 *	444	97	R11	2015-10-17	2015-10-21	R11	158	1
AJ12 *	422	95	R11	2015-10-17	2015-10-19	R11	192	1
AJ13	550	203.1	R16	2015-05-30	2015-06-01	R16	145	1
AJ14	511	159.3	R16	2015-05-30	2015-06-13	R15	48	2
AJ15 *	480	126	R16	2015-10-15	2015-11-17	R16	1	1
AJ16	513	153.3	R19	2015-05-30	2015-06-15	R19	87	1
AJ17	515	175.6	R19	2015-05-30	2015-06-13	R19	84	1
AJ18 *	620	305	R19	2015-10-15	2015-11-11	R18	134	2
AJ19 *	475	119	R19	2015-10-15	2015-10-30	R18	81	3

**Table 2:** Longitudinal and vertical movement distances of *A. japonica* (*, depth sensor; SD, standard deviation). Vertical movement distance is the sum of distances between each detection point.

ID	Detection Period (Day)	Longitudinal Movement (km)		Vertical Movement (m)			
		Movement Boundary	Total Distance	Nighttime		Daytime	
				Total	Average of One Day (SD)	Total	Average of One Day (SD)
AJ1	2	0	0	-	-	-	-
AJ2	10	0	0	-	-	-	-
AJ3	102	0	0	-	-	-	-
AJ4 *	21	0	0	0	0	0.4	0.4 (0)
AJ5 *	4	0	0	69.4	34.7 (11.7)	35.6	8.9 (6.8)
AJ6 *	6	0	0	138.8	34.7 (60.5)	1.0	1 (0)
AJ7 *	24	0	0	7.6	7.6 (0)	0	0
AJ8 *	30	24.2	72.6	59.2	29.6 (13.9)	0	0
AJ9 *	13	0	0	107.8	33.9 (14.9)	0	0
AJ10 *	4	0	0	198.8	49.7 (31.6)	66	22 (7.4)
AJ11 *	4	0	0	3.6	1.8 (1.4)	1.2	0.4 (0.2)
AJ12 *	2	0	0	13.2	4.4 (3.0)	9.2	4.6 (4.8)
AJ13	2	0	0	-	-	-	-
AJ14	14	5.6	5.6	-	-	-	-
AJ15 *	33	0	0	0	0	0	0
AJ16	16	0	0	-	-	-	-
AJ17	14	0	0	-	-	-	-
AJ18 *	27	6.5	6.5	77.4	19.4 (15.8)	0.8	0.8 (0)
AJ19 *	15	43	79.5	47	15.7 (14.4)	0	0
